# Stereocomplexation in Copolymer Networks Incorporating Enantiomeric Glycerol-Based 3-Armed Lactide Oligomers and a 2-Armed ɛ-Caprolactone Oligomer

**DOI:** 10.3390/ma9070591

**Published:** 2016-07-19

**Authors:** Ayaka Shibita, Seina Kawasaki, Toshiaki Shimasaki, Naozumi Teramoto, Mitsuhiro Shibata

**Affiliations:** Department of Life and Environmental Sciences, Faculty of Engineering, Chiba Institute of Technology, 2-17-1, Tsudanuma, Narashino, Chiba 275-0016, Japan; s1023130XU@s.chibakoudai.jp (A.S.); retastretassu@gmail.com (S.K.); shimasaki.toshiaki@it-chiba.ac.jp (T.S.); teramoto.naozumi@it-chiba.ac.jp (N.T.)

**Keywords:** star-shaped polymer, polymer network, crosslinking, stereocomplex, polylactide, glycerol, poly(ɛ-caprolactone)

## Abstract

The reactions of enantiomeric glycerol-based 3-armed lactide oligomers (H3DLAO and H3LLAO) and a diethylene glycol-based 2-armed ɛ-caprolactone oligomer (H2CLO) with hexamethylene diisocyanate (HDI) produced polyesterurethane copolymer networks (PEU-3scLAO/2CLOs 100/0, 75/25, 50/50, 25/75 and 0/100) with different feed ratios of stereocomplex (sc) lactide oligomer (H3scLAO = H3DLAO + H3LLAO, H3DLAO/H3LLAO = 1/1) and H2CLO. Thermal and mechanical properties of the copolymer networks were compared with those of a simple homochiral (hc) network (PEU-3DLAO) produced by the reaction of H3DLAO and HDI. X-ray diffraction and differential scanning calorimetric analyses revealed that sc crystallites are formed without any hc crystallization for PEU-3scLAO/2CLOs, and that PEU-3DLAO is amorphous. The melting temperatures of sc crystallites for PEU-3scLAO/2CLOs were much higher than that of hc crystallites of H3DLAO. The polarized optical microscopic analysis revealed that the nucleation efficiency is enhanced with increasing feed of H3scLAO fraction, whereas the spherulite growth rate is accelerated with increasing feed H2CLO fraction over 100/0-50/50 networks. PEU-3scLAO/2CLO 100/0 (i.e., PEU-3scLAO) exhibited a higher tensile strength and modulus than PEU-3DLAO. The elongation at break and tensile toughness for PEU-3scLAO/2CLOs increased with an increasing feed amount of H2CLO.

## 1. Introduction

Stereocomplex (sc) formation between enantiomeric poly(l-lactide) (PLLA) and poly(d-lactide) (PDLA) has garnered a great deal of attention because it is one of the most effective methods to enhance the properties of PLLA and PDLA [1,2,3,4,5,6,7,8,9]. The sc crystallites are reported to show a melting temperature (*T*_m_) approximately 50 °C higher than that of the homochiral (hc) crystallites [5]. Since the first report on sc-polylactide (PLA) by Ikada et al. [4], it has proven to be one of the most effective methods to enhance the properties of PLAs, including mechanical performance [7], thermal stability [6] and hydrolysis resistance [9]. It is known that a linear sc-PLA with a weight-average molecular weight (*M*_w_) not higher than 5 × 10^4^ has a high stereocomplexation ability, which is brittle, and that a linear sc-PLA with a *M*_w_ not lower than 1 × 10^5^ displays a higher tensile strength and modulus than the corresponding hc-PLA, while it has a relatively lower stereocomplexation ability, leading to the formation of hc crystallines along with sc crystallites [7]. Accordingly, a great deal of effort has been devoted to develop useful preparation methods, such as repeated casting [10], utilization of supercritical carbon dioxide [11] and annealing [12,13] for improving the sc efficiency. Recently, Bao et al. proposed a simple approach to generate pure sc-PLA by melt-blending PLLA and PDLA at low temperatures (160–210 °C), where only sc crystallites can grow [14,15]. However, sc-PLA has a weak melt memory to re-form sc crystallites after complete melting, and the ability to restore sc-PLA decreases with increasing molecular weight [16]. Therefore, sc-PLA derivatives, having a variety of macromolecular architectures, such as stereo-block copolymers having linear [17,18,19,20], cyclic [21], brush [22], and star-shaped [23,24,25] structures, and polymer blends having cyclic [26], comb-shaped [27], star-shaped [28,29,30,31,32,33,34,35,36,37,38,39,40,41,42] and dendritic [43] structures, have been applied to improve the melt-stability of sc-PLA. Regarding star-shaped sc-PLAs, Biela et al. reported an enhanced melt stability of star-shaped sc-PLA with 13-arms or more [28]. Isono et al. reported that solvent cast samples of 3-, 4- and 5-armed star shaped PLAs, having both PLLA and PDLA arms, formed sc-crystals without any homochiral crystallization [23]. Tsuji and Yamashita reported highly-accelerated sc crystallization by blending star-shaped 4-armed stereo diblock PLAs with PDLA and PLLA cores [40]. More recently, we reported that polyesterurethane copolymer networks (PEU-4scLAO/2CLOs) incorporating pentaerythritol-based enantiomeric 4-armed star-shaped lactide oligomers (H4LLAO and H4DLAO) and a diethylene glycol-based 2-armed ε-caprolactone oligomer (H2CLO) have excellent melt stability and tensile toughness [44]. The high melt stability is attributable to the fact that a melted l-lactide (LLA) oligomer segment is relatively close to the position of melted d-lactide (DLA) oligomer segment, because both the segments are incorporated in the polymer network. It is an interesting subject whether the high melt stability is reduced or not by changing the 4-armed enantiomeric lactide oligomers to 3-armed oligomers.

**Scheme 1:**
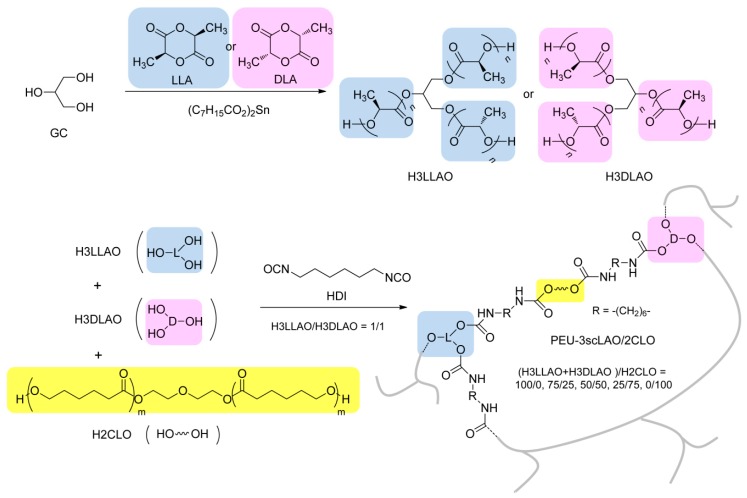
Synthesis route of PEU-3scLAO/2CLOs.

In this study, two enantiomeric 3-armed lactide oligomers (H3LLAO and H3DLAO) were prepared by the ring-opening polymerizations of LLA and DLA, initiated at the three hydroxy groups of glycerol, and crosslinked with H2CLO using hexamethylene diisocyanate (HDI) to produce polyesterurethane copolymer networks (PEU-3scLAO/2CLOs) with various feed ratios of stereocomplex lactide oligomer (H3scLAO, that is a mixture of equal parts of H3LLAO and H3DLAO) and H2CLO (Scheme 1). The influences of the feed ratio of (H3LLAO + H3DLAO)/H2CLO on the sc-formation, thermal and mechanical properties of the resulting networks were investigated. Our attention is focused on the stereocomplexation ability and tensile properties of PEU-3scLAO/2CLOs compared with those of PEU-4scLAO/2CLOs.

## 2. Experimental Section

### 2.1. Materials

Glycerol (GC) and 1,1,2-trichloroethane (TCE) were purchased from Kanto Chemical Co. Inc. (Tokyo, Japan). Hydroxy-terminated 2-armed ɛ-caprolactone oligomer (H2CLO) of which the catalogue name is polycaprolactone diol with a number-averaged molecular weight (*M*_n_) ~2000 was purchased from Sigma-Aldrich Corporation (St. Louis, MO, USA). The H2CLO is a diethylene glycol-initiated 2-armed ɛ-caprolactone oligomer with a degree of polymerization per arm of *m* = ca. 8.4 (Scheme 1). Hexamethylene diisocyanate (HDI) and ɛ-caprolactone (CL) were purchased from Tokyo Chemical Industry Co., Ltd. (Tokyo, Japan). Chlorobenzene was purchased from Wako Pure Chemical Industries, Ltd. (Osaka, Japan). Tin (II) bis(2-ethylhexanoate) (Sn(Oct)_2_) was purchased from KISHIDA CHEMICAL Co. Ltd. (Osaka, Japan). l-lactide (LLA, >99%) and d-lactide (DLA, >99%) were purchased from Musashino Chemical Laboratory, Ltd. (Tokyo, Japan). All the reagents were used without further purification.

### 2.2. Syntheses of H3LLAO and H3DLAO

A typical synthetic procedure of H3LLAO with a theoretical degree of polymerization of lactate unit (i.e., a half of lactide) per arm, *n* = 15, in this study is as follows: GC (0.378 g, 4.10 mmol), LLA (13.30 g, 92.28 mmol) and chlorobenzene (60 mL) were put into a nitrogen purged three-necked flask. The mixture was heated to 150 °C, and then Sn(Oct)_2_ (137 mg, 0.338 mmol) was added into the flask. The resulting mixture was stirred at 150 °C under a nitrogen atmosphere for 24 h. Hexane (500 mL) was added to the cooled reaction mixture with stirring, and then the supernatant was decanted off. This process was three times repeated and the separated precipitate was filtered, dried at 80 °C in a vacuum oven to give H3LLAO as a white powder (yield: 12.9 g, 94%). The degree of polymerization (*n*) per arm measured by ^1^H-NMR method was 17.8. H3DLAO was also synthesized as a white powder in 95% yield by the same procedure as that of H3LLAO except to use DLA instead of LLA. The value of *n* for H3DLAO was 15.2.

### 2.3. Synthesis of PEU-3scLAO/2CLO

A typical synthetic procedure of PEU-3scLAO/2CLO 75/25 is as follows: A solution of H3LLAO (1.05 g, 0.266 mmol), H3DLAO (1.04 g, 0.308 mmol), H2CLO (0.690 g, 0.345 mmol) and HDI (0.243 g, 1.45 mmol) in TCE (60 mL) was poured into a petri dish (diameter: 97 mm) made of poly(tetrafluoroethylene). The molar ratio of OH/NCO in the mixture was fixed to 1/1.2. The mixture was dried at 60 °C for 24 h, and then 130 °C for 4 h in an electric oven. The obtained PEU-3scLAO/2CLO with the feed weight ratio of (H3LLAO + H3DLAO)/H2CLO 75/25 (thickness: ca. 0.3 mm) was peeled off from the petri dish. PEU-3scLAO/2CLO (100/0, 50/50, 25/75 and 0/100) films were also prepared by a similar method. PEU-3scLAO/2CLO 100/0 and PEU-3scLAO/2CLO 0/100 were also abbreviated as PEU-3scLAO and PEU-2CLO, respectively. The reaction products of H3DLAO with HDI (PEU-3LLAO) and a stereocomplex of H3LLAO and H3DLAO (H3scLAO) were also prepared by a similar method for comparison.

### 2.4. Characterization and Measurements

Proton nuclear magnetic resonance (^1^H-NMR) spectra were recorded on a Bruker AV-400 (400 MHz) (Madison, WI, USA) using CDCl_3_ and tetramethylsilane as a solvent and an internal standard, respectively.

Fourier transform infrared (FT-IR) spectra were recorded at room temperature in the range from 4000 to 700 cm^−1^ on a Shimadzu (Kyoto, Japan) FT-IR 8400s by the attenuated total reflectance (ATR) method. The IR spectra were acquired using 50 scans at a resolution of 4 cm^−1^. Degree of swelling (*D*_s_) was measured by dipping a film (10 × 5 × 0.3 mm^3^) in chloroform, *N*,*N*-dimethylformamide (DMF) or ethanol at room temperature for 2 days according to the following equation: $D_{s}(\text{wt }\%)=100(w_{1}-w_{0})/w_{0}$, where *w*_0_ is the initial weight of the film and *w*_1_ is the weight of the swollen film after dipping. In addition, the film dipped in chloroform for 3 days was dried at 40 °C in a vacuum oven for 24 h, and the weight reduction percentage by the chloroform extraction was calculated by the equation: Weight reduction percentage (%) = $100(w_{0}-w_{2})/w_{0}$; where *w*_2_ is the weight of the dried film.

X-ray diffraction (XRD) analysis was performed at ambient temperature on a Rigaku (Tokyo, Japan) RINT-2100 X-ray diffractometer at a scanning rate of 2.0° min^−1^, using Cu Kα radiation (wavelength, λ = 0.154 nm) at 40 kV and 14 mA. All scans were in the range 5° ≤ 2θ ≤ 30° at a scanning rate of 1.0° min^−1^ and a step size of 0.01°.

Differential scanning calorimetry (DSC) measurements were performed on a Perkin-Elmer (Waltham, MA, USA) Diamond DSC in a nitrogen atmosphere. The as-prepared samples (8–12 mg) were heated from −100 °C to 200 °C at a heating rate of 20 °C·min^−1^, held at the temperature for 3 min to eliminate a thermal history of the sample, and then cooled to −100 °C at a cooling rate of 100 °C·min^−1^. After being held at −100 °C for 3 min, the second heating scan was monitored at a heating rate of 20 °C·min^−1^. Glass transition temperature (*T*_g,*x*_), cold crystallization temperature (*T*_c,*x*_), enthalpy of cold crystallization (∆*H*_c,*x*_), melting temperature (*T*_m,*x*_) and enthalpy of melting (∆*H*_m,*x*_) for each component (*x* = LAO or CLO) were determined from the first and second heating curves. The crystallinities (χ_c,*x*_s) of hc or sc oligolactide crystallites and oligocaprolactone crystallites were calculated using the following equation:

$$\chi_{\text{c},x}(\%)=\left(\frac{\Delta H_{\text{m},x}}{w\Delta H_{\text{m},x}^{0}}\right)\times 100$$

where *w* is the weight fraction of (H3LLAO + H3DLAO) and $\Delta H_{\text{m},x}^{0}$ is enthalpy of 100% crystalline hc-PLA (93 J·g^−1^) or sc-PLA (142 J·g^−1^) in case of *x* = LAO [5,23], and *w* is the weight fraction of H2CLO and $\Delta H_{\text{m},x}^{0}$ is enthalpy of 100% crystalline PCL (139 J·g^−1^) in case of *x* = CLO [45].

Morphology of fractured surfaces of the copolymer networks was observed by field emission-scanning electron microscopy (FE-SEM), using a Hitachi S-4700 machine (Hitachi High-Technologies Corporation, Tokyo, Japan). All samples were fractured after immersion in liquid nitrogen for about 5 min. The fracture surfaces were sputter-coated with gold to provide enhanced conductivity.

Polarized optical microscopy was performed on an Olympus BXP polarizing microscope equipped with a Japan High-tech hot-stage RH-350 and a Sony CCD-IRIS color video camera. After a sample was heated to 220 °C on the hot-stage and held at 220 °C for 10 min, it was cooled to a specified temperature (120–150 °C) at a cooling rate of 50 °C·min^−1^ and the growing of spherulites was monitored over time at that temperature.

Dynamic mechanical analysis (DMA) of the rectangular plates (40 × 8 × 0.3 mm^3^) was performed on a Rheolograph Solid instrument (Toyo Seiki Co., Ltd., Tokyo, Japan) under an atmosphere of air with a chuck distance of 20 mm, a frequency of 1 Hz and a heating rate of 2 °C·min^−1^, based on ISO 6721-4:1994 (Plastics-Determination of dynamic mechanical properties, Part 4: Tensile vibration−Non-resonance method).

The 5% weight loss temperature (*T*_5_) was measured on a Shimadzu TGA-50 thermogravimetric analyzer. A sample of about 5 mg was heated from room temperature to 500 °C at a heating rate of 20 °C·min^−1^ in a nitrogen purge stream at a flow rate of 50 mL·min^−1^.

Tensile testing of rectangular specimens (length 45 mm, width 7 mm, thickness 0.3 mm) was performed at 25 °C using a Shimadzu Autograph AG-1, based on the standard method for testing the tensile properties of plastics (JIS K7161:1994 (ISO527-1:1993)). Span length and testing speed were 25 mm and 3 mm·min^−1^, respectively. Five specimens were tested for each set of samples, and the mean values and the standard deviation were calculated.

## 3. Results and Discussion

### 3.1. Polymer Network Formation of PEU-3scLAO/2CLOs

**Figure 1:**
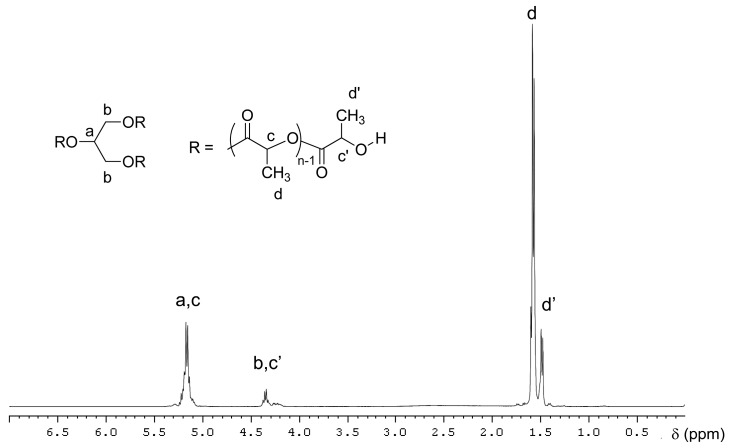
^1^H-NMR spectrum of H3LLAO in CDCl_3_.

H3LLAO or H3DLAO were synthesized by the ring-opening polymerization of LLA or DLA initiated at three hydroxy groups of GC at a lactide/hydroxy ratio of 7.5 (Scheme 1). Figure 1 shows ^1^H-NMR spectrum of H3LLAO in CDCl_3_. Methine proton signals (H^c^ and H^c′^) of repeating and terminal lactate units of H3LLAO were separately observed at δ 5.18 and 4.36 ppm, respectively. The two signals overlapped with the methine (H^a^) and methylene (H^b^) proton signals of the GC moiety, respectively. Additionally, methyl proton signals (H^d^ and H^d′^) of repeating and terminal lactate units of H3LLAO were closely observed at δ 1.59 and 1.49 ppm, respectively. The degree of polymerization (*n*) of lactate unit (i.e., half of lactide) per arm for H3LLAO was calculated to be 17.8, based on the integral ratio of H^a,c^/H^b,c′^. The *n* value measured by the same ^1^H-NMR method for H3DLAO was 15.2. Although the *n* value of H3LLAO was a slightly higher than that of H3DLAO, the difference should not be attributed to a difference of LLA and DLA but to experimental error, because the *n* value evaluated by ^1^H-NMR method deviates by ca. 15% in several experiments. If the *n* values of H3LLAO and H3DLAO were lower than 15, the stereocomplexation ability becomes worse [44]. Conversely, if the *n* values were much higher than 15, the subsequent crosslinking reaction becomes difficult because of a much lower terminal hydroxy content. The molecular weights calculated from the *n* values (17.8 and 15.2) for H3LLAO and H3DLAO were 3940 and 3378, respectively. These values were used for calculation of their feed amounts for the reaction with HDI. PEU-3scLAO/2CLO films were prepared by the crosslinking reaction of H3LLAO, H3DLAO, H2CLO and HDI (Scheme 1). In addition, an uncrosslinked sc-H3LLAO/H3DLAO blend (H3scLAO) was prepared for comparison. As the H3scLAO was obtained as a white powder, the formation of network structure was very important to produce a tough film. In addition, the crosslinking by urethane linkages is important to not spoil the biodegradable and biocompatible properties of the polyester segments [46,47].

**Figure 2:**
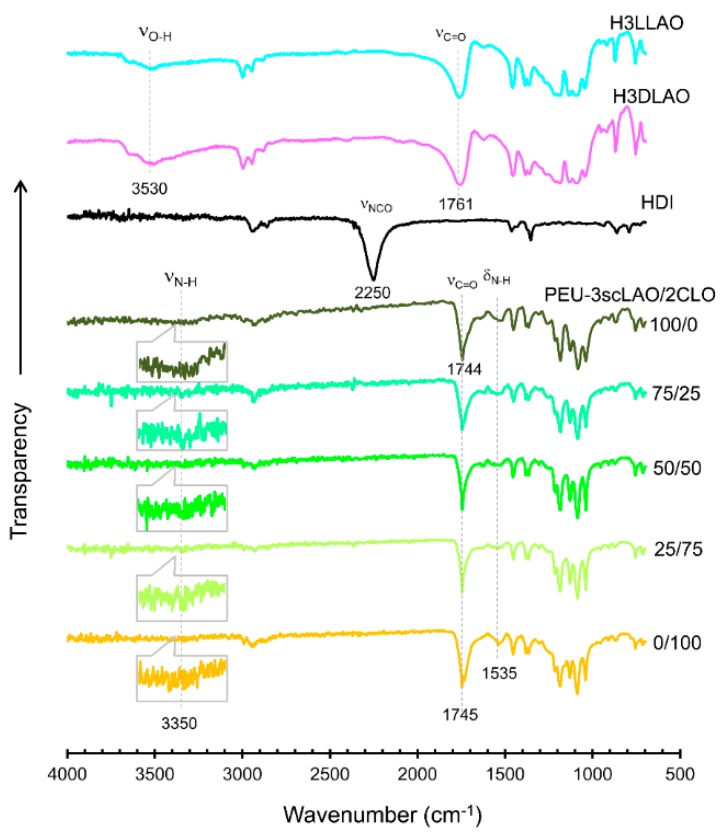
FT-IR spectra of H3LLAO, H3DLAO, PEU-3scLAO/2CLOs (100/0, 75/25, 50/50, 25/75 and 0/100) and HDI. For PEU-3scLAO/2CLOs, longitudinally enlarged spectra at the wavelength region from 3100 to 3600 cm^−1^ are also shown in the figure.

Figure 2 shows FT-IR spectra of PEU-3scLAO/2CLOs compared with those of H3LLAO, H3DLAO and HDI. The FT-IR spectrum of H3LLAO was substantially similar to that of H3DLAO. The weak band at 3530 cm^−1^ due to O–H stretching vibration (ν_O–H_) observed for H3LLAO and the strong band due to NCO stretching vibration (ν_NCO_) at 2250 cm^−1^ observed for HDI were almost nonexistent for PEU-3scLAO/2CLOs. Although absorption bands characteristic of urethane bond for PEU-3scLAO/2CLOs are not so strong because the fraction of urethane bonds formed in the polymer network is slight, new absorption bands of N−H stretching vibration (ν_N–H_) and N−H bending vibration (δ_N−H_) certainly appeared at 3350 and 1535 cm^−1^, respectively. The band of urethane C=O stretching vibration (ν_C=O_) overlapped with the ester ν_C=O_ band of the lactide oligomer (LAO) and caprolactone oligomer (CLO) segments of PEU-3scLAO/2CLOs at ca. 1745 cm^−1^. These results suggest that the reaction of hydroxy group of H3LLAO/H3DLAO and isocyanate group of HDI proceeded smoothly to generate a polymer network having bridged urethane linkages.

**Figure 3:**
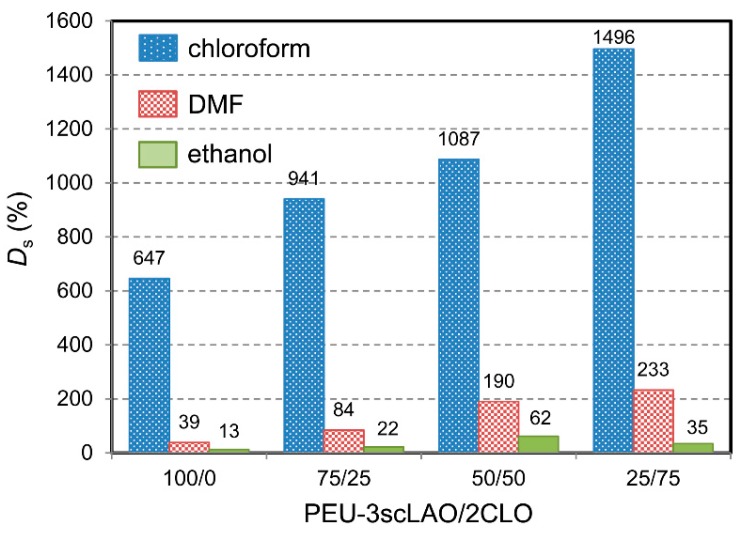
Degree of swelling for PEU-3scLAO/2CLOs (100/0, 75/25, 50/50, 25/75 and 0/100) in chloroform, DMF or ethanol.

The formation of polymer networks for PEU-3scLAO/2CLOs (100/0, 75/25, 50/50 and 25/75) were also confirmed by swelling and extraction experiments. Figure 3 shows *D*_s_ values of PEU-3scLAO/2CLOs dipped in chloroform, DMF or ethanol for 2 days. The *D*_s_ increased with increasing CLO fraction, indicating that crosslinking density decreases with increasing feed of 2-armed H2CLO. In the case of using ethanol, the *D*_s_ values (13%–62%) in ethanol were much lower than those in chloroform and DMF, suggesting that the network has a low affinity toward ethanol. Furthermore, the weight reduction percentages of PEU-3scLAO/2CLOs (100/0, 75/25, 50/50 and 25/75) after extracted with chloroform for 3 days were 1.64%, 2.51%, 2.39% and 16.7%, respectively. The fact that 25/75 network had a slightly higher weight reduction percentage than the other networks should be caused by the extraction of branched polymers, with a higher fraction of H2CLO-based moiety. PEU-3scLAO/2CLO 0/100 (i.e., PEU-2CLO) completely dissolved in chloroform because the sample is a linear polymer.

### 3.2. Stereocomplex Formation in PEU-3scLAO/2CLOs

**Figure 4:**
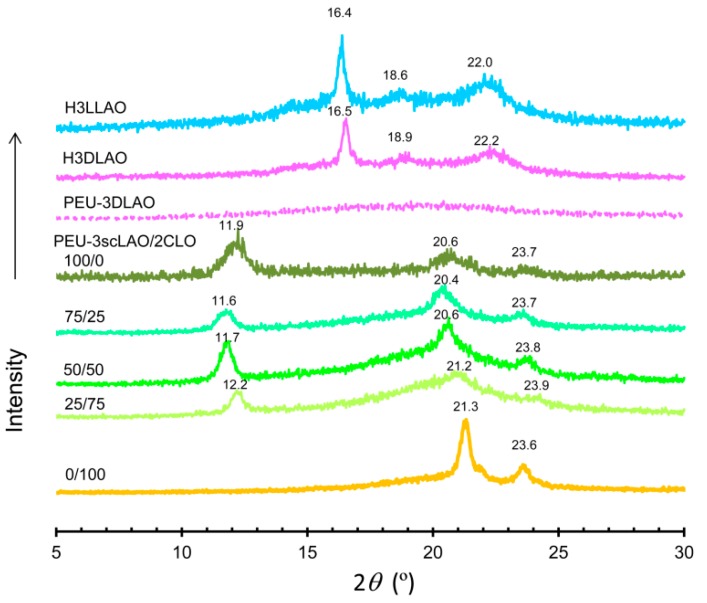
XRD profiles of H3LLAO, H3DLAO, PEU-3DLAO and PEU-3scLAO/2CLOs (100/0, 75/25, 50/50, 25/75 and 0/100).

Figure 4 shows XRD profiles of H3LLAO, H3DLAO, PEU-3DLAO and PEU-3scLAO/2CLOs. The XRD patterns of H3LLAO and H3DLAO exhibited diffraction peaks of hc crystals at 2θ values of 16.4°–16.5°, 18.6°–18.9° and 22.0°–22.2° in a similar manner to PLLA and PDLA [5,6,48]. The peaks characteristic of hc crystallines did not appear for PEU-3DLAO, suggesting that the formation of a polymer network by urethane linkages prevented the hc crystallization. A similar suppression of crystallization of hc LAO chains by crosslinking was observed for the polymer network prepared by the crosslinking reaction of H4LLAO or H4DLAO with methylenediphenyl 4,4′-diisocyanate [44]. PEU-3scLAO/2CLO exhibited diffraction peaks at 2θ values of 11.6°–12.2°, 20.4°–21.2° and 23.7°–23.8°, characteristic of sc crystallites [5,49]. No diffraction peaks due to the hc crystallites were observed in the XRD profiles of PEU-3scLAO/2CLOs, suggesting that sc crystallites were selectively formed without the formation of hc crystallites. A similar result was also observed for the previously reported PEU-4scLAO/2CLO [44]. PEU-3scLAO/2CLO 0/100 (i.e., PEU-2CLO) displayed diffraction peaks at 2θ values at 21.3° and 23.6° corresponds to the (001) and (200) planes of oligocaprolactone (CLO) crystallites, respectively [50]. The XRD profile of PEU-3scLAO/2CLO 25/75 exhibited diffraction peaks ascribed to both sc-LAO and CLO crystallites. However, we could not determine the precise crystallinities of the sc-LAO and CLO crystals in PEU-3scLAO/2CLOs 75/25, 50/50 and 25/75, because their XRD peaks are closely observed.

Table 1 summarizes the data estimated from the first heating DSC curves for H3DLAO, PEU-3DLAO, H3scLAO and PEU-3scLAO/2CLOs (100/0, 75/25, 50/50, 25/75 and 0/100). The first heating DSC curves are also shown in Figure S1 of the Supplementary Materials. H3DLAO exhibited a *T*_g,LAO_ and a *T*_m,LAO_ of hc crystals at 59.9 °C and 118.0 °C, respectively. PEU-3DLAO showed only a *T*_g,LAO_ at 64.7 °C, whose value was higher than that of H3DLAO. This result suggests that the formation of a polymer network disturbed the chain mobility. The fact that no *T*_m,LAO_ was observed for PEU-3DLAO indicates that the hc polymer networks are amorphous, which in agreement with the results of XRD. Among the as-prepared samples, only H3scLAO displayed a *T*_c,LAO_ at 88.9 °C (∆*H*_c,LAO_ = −31.2 J·g^−1^), implying that the sc-crystallization was not completed during the sample preparation. Considering that the as-prepared H3scLAO is a powder sample, it is supposed that imperfect sc-crystallites are precipitated after mixing of H3LLAO and H3DLAO in TCE. PEU-3scLAO/2CLOs (100/0, 75/25, 50/50 and 25/75) displayed the *T*_m,LAO_ at 173.1–178.5 °C, which were much higher than the *T*_m,LAO_ (118.0 °C) of H3DLAO, and were a little lower than the *T*_m,LAO_ (186.5 °C) based on sc crystals of H3scLAO. The χ_c,LAO_s (24%–30%) of sc-crystallites for PEU-3scLAO/2CLOs (100/0, 75/25, 50/50 and 25/75) were comparable to those (28.2%) for H3scLAO. The χ_c,LAO_ for PEU-3scLAO/2CLOs did not largely reduce with an increasing CLO fraction, indicating that sc crystallization is not totally disturbed by the presence of CLO moiety. Additionally, the *T*_m,LAO_ (173.1–178.5 °C) for PEU-3scLAO/2CLOs was a little lower than the already-reported *T*_m,LAO_ (179.5–180.3 °C) for PEU-4scLAO/2CLOs [44]. This result may be attributable to a possibility that the three arm lengths of H3LLAO (or H3DLAO) are not the same, but those of H4LLAO (or H4DLAO) are almost the same, considering that the reactivity of hydroxy groups adjacent to methylene and methine in glycerol, which was used as a core molecule of the former, vary from each other, and that the reactivity of hydroxy groups in pentaerythritol for the latter does not vary. On the other hand, the χ_c,LAO_ (23.9%–29.6%) for PEU-3scLAO/2CLOs was a little higher than the already-reported χ_c,LAO_ (12.1%–22.5%) for PEU-4scLAO/2CLOs, suggesting that a lower crosslinking density for PEU-3scLAO/2CLOs leads to a higher chain mobility, facilitating the crystallization. We could not observe clear *T*_g,LAO_s in the first heating DSC thermograms of PEU-3scLAO/2CLOs (75/25, 50/50 and 25/75).

Table 2 summarizes the data evaluated from the second heating DSC curves of all the samples after cooling at a rate of 100 °C·min^−1^ from 200 °C to −100 °C. The second heating DSC curves are also shown in Figure S2 of the Supplementary Materials. The fact that the values of ∆*H*_c,LAO_ and ∆*H*_m,LAO_ were close to each other indicates that the sample before the second heating is almost amorphous. The *T*_c,LAO_s and *T*_m,LAO_s of PEU-3scLAO/2CLOs were observed at 69–92 °C and 175–183 °C, respectively. The *T*_m,LAO_s estimated from the second heating scans were comparable to those (173–179 °C) from the first heating scans, indicating that almost the same sc-crystallites are regenerated after melting at 200 °C. It is noteworthy that PEU-3scLAO/2CLOs (100/0, 75/25, 50/50 and 25/75) showed no *T*_c,LAO_s and *T*_m,LAO_s based on hc crystallites. This is probably caused by the possibility that a melted LLA oligomer segment is relatively close to the position of a melted DLA oligomer segment, because both the segments are incorporated in the polymer network. The *T*_m,LAO_ (176.6 °C) and χ_c,LAO_ (16.4%) for PEU-3scLAO/2CLO 100/0 were lower than those (186.5 °C and 26.6%) of H3scLAO, respectively, implying that the urethane crosslinking disturbed the sc crystallization to some extent. The fact that the χ_c,LAO_ of PEU-3scLAO/2CLO 75/25 was higher than that of the 100/0 sample indicates that the incorporation of CLO segments enhanced cold crystallization of LAO segments.

**Figure 5:**
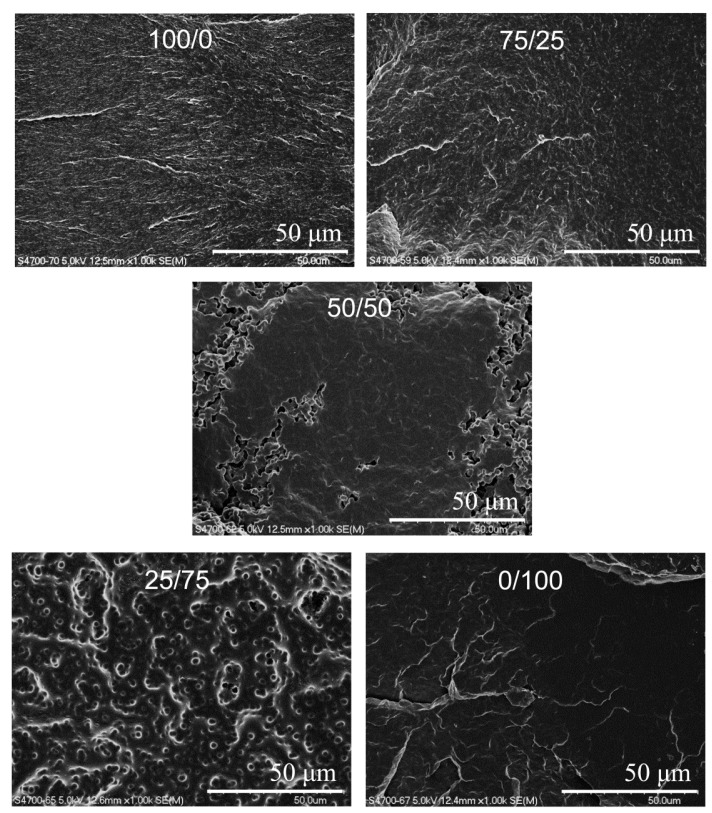
FE-SEM images of the fractured surfaces of PEU-3scLAO/2CLOs (100/0, 75/25, 50/50, 25/75 and 0/100).

Figure 5 shows FE-SEM images of the fractured surfaces of PEU-3scLAO/2CLOs. Although the surface of PEU-3scLAO/2CLO 100/0 (i.e., PEU-3scLAO) was rough, probably because of the formation of sc-crystallites, it was homogeneous in a similar manner to that of PEU-2CLO. The compatibility between the LAO and CLO components for PEU-3scLAO/2CLO 75/25 is better than that for PEU-3scLAO/2CLOs 50/50 and 25/75, as is obvious from the presence of many domains having a size of several micron (μm) for the latter. It is supposed that the reaction of H3LLAO, H3DLAO, H2CLO and HDI was not completed in a homogeneous TCE solution, and 3scLAO-rich branched polymers phase-separated from 2CLO-rich branched polymers after evaporation of TCE for the 50/50 and 25/75 networks. It is considered that several holes for the 25/75 sample are caused by the pull-out of LAO domains during the fracturing of the sample.

**Figure 6:**
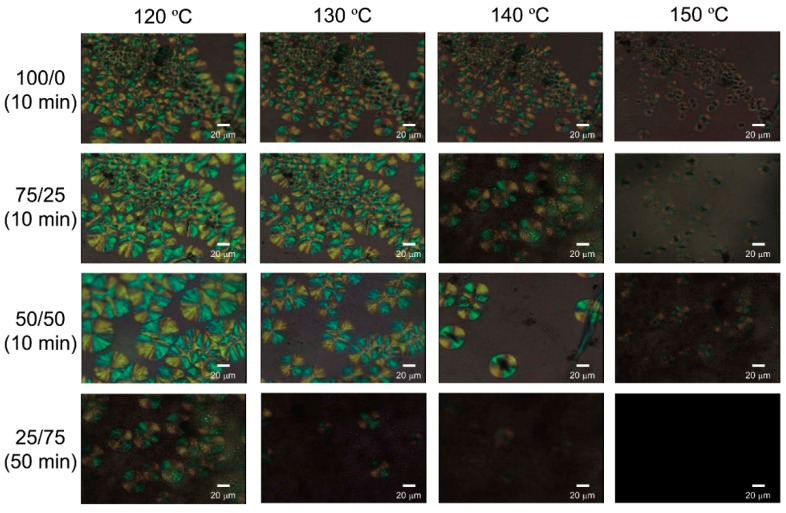
Polarized optical microscope images of the PEU-3scLAO/2CLOs (100/0, 75/25, 50/50, 25/75 and 0/100) held at a specified temperature for 10 or 50 min after melted at 220 °C.

Figure 6 shows polarized optical microscope images of the PEU-3scLAO/2CLOs, which were independently held at a specified temperature (120, 130, 140 or 150 °C) after being melted at 220 °C. The holding time at each crystallization temperature was 10 min for PEU-3scLAO/2CLOs 100/0, 75/25 and 50/50. As no crystallites were observed at a holding time of 10 min for PEU-3scLAO/2CLO 25/75, the photographs at a holding time of 50 min were shown in the figure. For all of the PEU-3scLAO/2CLOs, the crystallization temperature where the largest total volume of sc-LAO crystallites are formed was 120 °C, and the sc-LAO crystallization was suppressed with increasing temperature until 150 °C. Regarding the effect of the LAO/CLO ratio, the largest number of sc-LAO spherulites was formed for the 100/0 sample, and the number decreased with increasing CLO fraction. On the other hand, sc-LAO spherulites with the biggest size were formed for the 50/50 sample, and the size was reduced with increasing LAO fraction. In case of the 25/75 sample, the nucleation rate is very slow, and the size of spherulites after 50 min was comparable to that for 75/25. These results indicate that the nucleation efficiency is enhanced with increasing LAO fraction, and the spherulite growth rate is accelerated with increasing CLO fraction over 100/0–50/50. The incorporating of flexible CLO fraction may facilitate the movement of LAO segments to sc-LAO crystalline growth edges. Regarding the enhancement of nucleation efficiency, the encounter between LLAO and DLAO segments may be made easier with decreasing CLO fraction.

### 3.3. Thermal and Mechanical Properties of PEU-3scLAO/2CLOs

**Figure 7:**
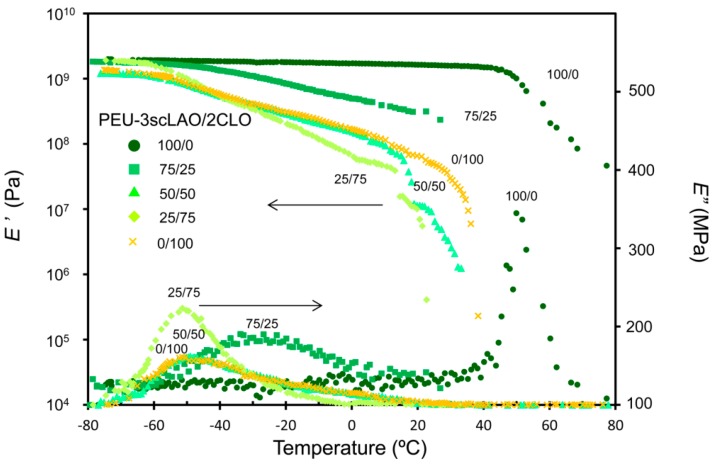
DMA curves of PEU-3scLAO/2CLOs (100/0, 75/25, 50/50, 25/75 and 0/100).

Figure 7 shows DMA curves of PEU-3scLAO/2CLOs (100/0, 75/25, 50/50, 25/75 and 0/100). The peak temperatures of loss moduli (*E*″s) corresponding to *T*_g_s for PEU-3scLAO and PEU-2CLO were 51.7 and −51.4 °C. The *E*″ peak temperature (−26.8 °C) for PEU-3scLAO/2CLO 75/25 was much higher than that for PEU-2CLO, suggesting that the LAO and CLO segments are compatibilized in agreement with the results of FE-SEM. The *E*″ peak temperatures (−49.0 and −51.4 °C) for CLO-rich phase of PEU-3scLAO/2CLOs 50/50 and 25/75 were close to that (−51.4 °C) of PEU-2CLO, whereas we could not observe the *E*″ peak temperatures related to the LAO-rich phase because the DMA measurements stopped at around 40 °C due to melting of the CLO segment, suggesting that the CLO segments are phase-separated, in agreement with the FE-SEM. The storage modulus (*E*′) of PEU-3scLAO dropped at around 45 °C due to a glass transition of the LAO-segment. The *E*′ of PEU-2CLO was reduced at around −50 °C due to a glass transition of the CLO segment, and dropped at around 30 °C due to melting of the CLO segment. The fact that PEU-3scLAO/2CLOs 50/50 and 25/75 exhibited similar *E*′ curves to PEU-2CLO reflects that the CLO segments are phase-separated. On the other hand, when the *E*′ values at the same temperature are compared, the *E*′ of PEU-3scLAO/2CLO 75/25 was lower than that of PEU-3scLAO, and higher than that of PEU-2CLO, in agreement with the fact that the LAO and CLO segments in PEU-3scLAO/2CLO 75/25 are compatibilized.

**Figure 8:**
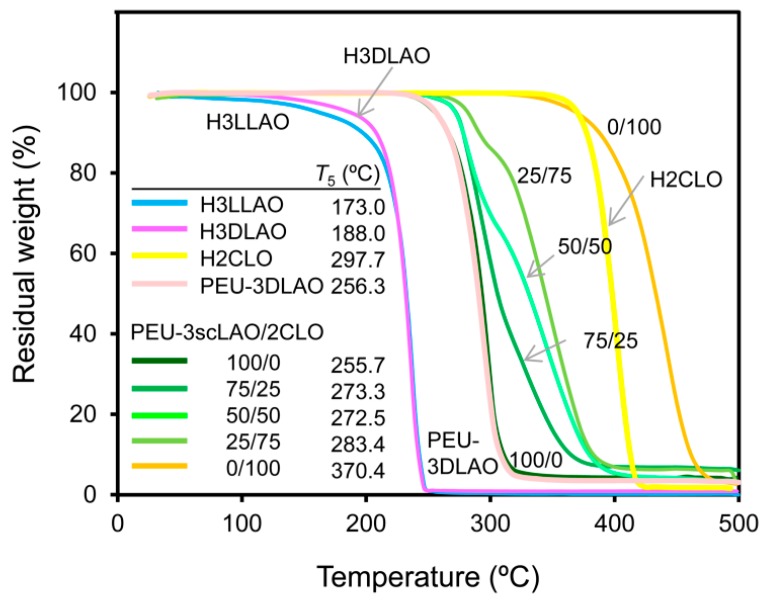
TGA curves of H3LLAO, H3DLAO, H2CLO, PEU-3DLAO and PEU-3scLAO/2CLOs (100/0, 75/25, 50/50, 25/75 and 0/100).

Figure 8 shows TGA curves of H3LLAO, H3DLAO, H2CLO, PEU-3DLAO and PEU-3scLAO/2CLOs (100/0, 75/25, 50/50, 25/75 and 0/100). The *T*_5_ for PEU-3scLAO/2CLOs increased with increasing CLO fraction in agreement with the fact that the *T*_5_ (297.7 °C) of H2CLO was much higher than those (173.0 and 188.0 °C) of H3LLAO and H3DLAO. There is little difference in *T*_5_ between PEU-3DLAO and PEU-3scLAO in agreement with the fact that there is little difference in thermal stability between sc-PLA and PLLA or PDLA [8]. PEU-3scLAO/2CLOs (75/25, 50/50 and 25/75) showed two-step thermodegradation curves, which are caused by the decomposition of LAO and CLO components.

**Figure 9:**
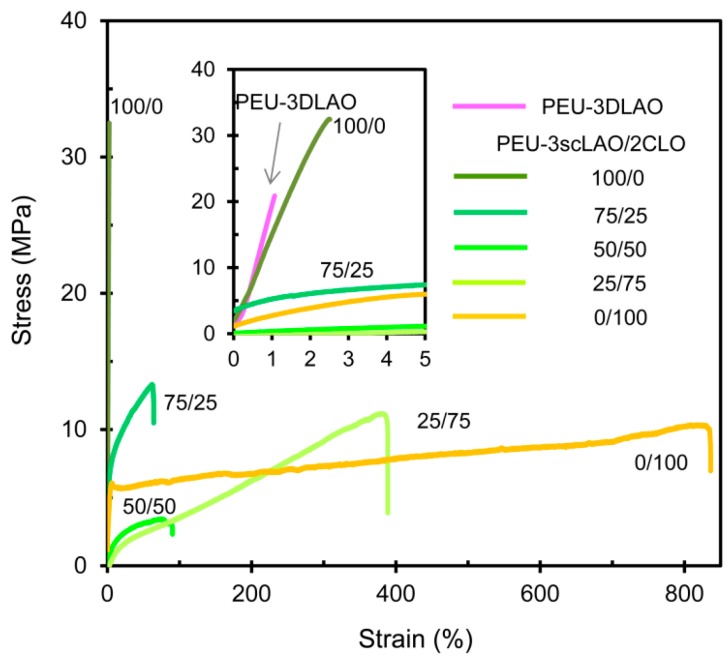
Stress-strain curves of PEU-3scLAO/2CLOs (100/0, 75/25, 50/50, 25/75 and 0/100).

Figure 9 shows typical stress-strain curves of PEU-3DLAO and PEU-3scLAO/2CLOs (100/0, 75/25, 50/50, 25/75 and 0/100). Strain (elongation) at break and initial slope (tensile modulus) for PEU-3scLAO were much lower and higher than those of PEU-2CLO, respectively, in agreement with the fact that LAO segment is much stiffer than CLO segment. Maximal stress and tensile modulus for PEU-3scLAO/2CLOs decreased, and elongation at break inversely increased with increasing CLO fraction. As the toughness is defined as the energy needed to break a sample of unit area and unit length (J·m^−3^), it is given by the area under the stress-strain curve [44]. From the stress-strain curves shown in Figure 9, it is obvious that the toughnesses of PEU-3scLAO/2CLOs (75/25, 50/50, 25/75 and 0/100) are much higher than that of PEU-3scLAO. The maximal stress of PEU-3scLAO was certainly higher than those of PEU-3DLAO, as is shown in the enlarged graph of Figure 9. The fact that the maximal stress of PEU-3scLAO/2CLO 50/50 was lower than that of the 25/75 sample may be related to the phase-separated morphology and/or low χ_c,CLO_ of the 50/50 sample (Figure 5 and Table 1).

Table 3 summarizes tensile strength, modulus and elongation at break in addition to tensile toughness, which was calculated from the area of a stress-strain curve. Tensile strength (54.7 MPa), tensile (Young’s) modulus (2.37 GPa), and elongation at break (2.98%) of PEU-3scLAO were a little higher than those (42.9 MPa, 2.21 GPa, and 2.26%) of PEU-3DLAO. These results are in agreement with the fact that a linear sc-PLA film with *M*_w_ from 1 × 10^5^ to 1 × 10^6^ surpassed the corresponding PLLA or PDLA films in tensile strength, Young’s (tensile) modulus and elongation at break [5,7]. It is noteworthy that the tensile strength and modulus of PEU-3scLAO are much higher than those (45 MPa and 1820 MPa) of sc-PLA with *M*_w_ 1.3 × 10^6^ [5] and those (34.7 MPa and 1695 MPa) of PEU-4scLAO/2CLO 100/0 (i.e., PEU-4scLAO) [44]. Although tensile strength and modulus of PEU-3scLAO/2CLO decreased with increasing CLO fraction, elongation at break and tensile toughness significantly increased with CLO fraction. It is known that a linear sc-PLA with a *M*_w_ not higher than 5 × 10^4^ has a high stereocomplexation ability, which is brittle, and that a linear sc-PLA with a *M*_w_ not lower than 1 × 10^5^ displays a higher tensile strength and modulus than the corresponding hc-PLA does, while it has a relatively lower stereocomplexation ability [7]. In a similar manner, although H3scLAO with a molecular weight of ca. 4000 had a high sc-LAO crystallinity, it was a powder sample, which hardly ever becomes a tough film. The macromolecular architecture for PEU-3scLAO/2CLOs is effective to prepare tough films from 3-armed LLA and DLA oligomers, which have a high stereocomplexation ability. As the crosslinked resins in this study are not suitable for the material recycling, and are expected to be used as biomaterials, we plan to investigate the biocompatibility and biodegradability behavior in the future.

## 4. Conclusions

The stereocomplex formation, thermal and mechanical properties of PEU-3scLAO/2CLOs (100/0, 75/25, 50/50, 25/75 and 0/100) prepared by reactions of H3LLAO, H3DLAO and H2CLO with HDI were compared with those of H3scLAO, PEU-3DLAO. Although H3scLAO was obtained as a powder sample, PEU-3scLAO/2CLOs and PEU-3DLAO became relatively tough films. The formation of network structure by reactions of the hydroxy and isocyanate groups for PEU-3scLAO/2CLOs was confirmed by the FT-IR analysis, extraction and swelling experiments using chloroform. XRD analysis revealed that sc crystallites are preferentially formed for PEU-3scLAO/2CLOs and H3scLAO, while hc crystallites are formed for H3LLAO and H3DLAO, and that PEU-3DLAO is amorphous. The melting temperatures of sc crystallites for PEU-3scLAO/2CLOs were much higher than those of hc crystallites of H3LLAO and H3DLAO, while the values were slightly lower than that of H3scLAO. Although cold crystallization temperatures for PEU-3scLAO/2CLOs were not observed during the first heating scan, but the temperatures were observed during the second heating scans after cooling at a rate of 100 °C·min^−1^ from 200 °C. No melting temperatures of hc crystallites were observed for PEU-3scLAO/2CLOs in the first and second heating DSC curves. The polarized optical microscopic analysis revealed that the nucleation efficiency was enhanced with increasing LAO fraction, whereas the spherulite growth rate was accelerated with increasing CLO fraction over 100/0–50/50 networks. The change of *E*″ peak temperatures measured by DMA was in agreement with the compatibilizaion for PEU-3scLAO/2CLO 75/25 and phase-separated morphology of PEU-3scLAO/2CLOs 50/50 and 25/75. Tensile strength, modulus and elongation at break of PEU-3scLAO were higher than those of PEU-3DLAO, sc-PLA and PEU-4scLAO. Elongation at break and tensile toughness for PEU-3scLAO/2CLOs increased with increasing CLO fraction. It was noteworthy that the PEU-3scLAO based on 3-armed lactide oligomers exhibited much better tensile properties than the PEU-4scLAO based on 4-armed lactide oligomers without any reduction of the high stereocomplexation ability and melt stability. The sc copolymer networks in this study are expected to be used as biodegradable and biocompatible materials requiring better tensile properties than those of PLA or sc-PLA.

## Figures and Tables

**Table 1:** Data collected from the first heating DSC curves of H3DLAO, PEU-3DLAO, H3scLAO and PEU-3scLAO/2CLOs (100/0, 75/25, 50/50, 25/75 and 0/100).

Sample	*T*_g,LAO_ (°C)	*T*_m,CLO_ (°C)	∆*H*_m,CLO_ (J·g^−1^)	χ_c,C/LO_ (%)	*T*_c,LAO_ (°C)	∆*H*_c,LAO_ (J·g^−1^)	*T*_m,LAO_ (°C)	∆*H*_m,LAO_ (J·g^−1^)	χ_c,LAO_ (%)
H3DLAO	59.9	-	-	-	-	0	118.0	22.2	23.9
PEU-3DLAO	64.7	-	-	-	-	0	-	0	0
H3scLAO	45.3	-	-	-	88.9	–31.2	186.5	40.1	28.2
PEU-3scLAO/2CLO									
100/0	65.9	-	-	-	-	0	178.1	31.5	23.8
75/25	-	-	0	0	-	0	173.1	27.4	27.7
50/50	(–45.2) ^1^	−31.7	1.67	2.6	-	0	176.5	19.4	29.6
25/75	(–45.5) ^1^	36.0	7.72	8.0	-	0	178.5	8.46	25.6
0/100	-	53.1	30.5	23.8	-	-	-	-	-

^1^ *T*_g,CLO_.

**Table 2:** Data collected from the second heating DSC curves of H3DLAO, PEU-3DLAO, H3scLAO and PEU-3scLAO/2CLOs (100/0, 75/25, 50/50, 25/75 and 0/100).

Sample	*T*_g,LAO_ (°C)	*T*_m,CLO_ (°C)	∆*H*_m,CLO_ (J·g^−1^)	χ_c,CLO_ (%)	*T*_c,LAO_ (°C)	∆*H*_c,LAO_ (J·g^−1^)	*T*_m,LAO_ (°C)	∆*H*_m,LAO_ (J·g^−1^)	χ_c,LAO_ (%)
H3DLAO	35.0	-	-	-	-	0	-	0	0
PEU-3DLAO	58.9	-	-	-	-	0	-	0	0
H3scLAO	47.8	-	-	-	88.9	−31.6	186.5	37.8	26.6
PEU-3scLAO/2CLO									
100/0	42.3	-	-	-	90.4	−25.9	176.6	21.7	16.4
75/25	-	-	0	0	68.5	−21.0	176.9	21.1	21.3
50/50	56.9	-	0	0	91.8	−12.9	174.6	12.9	19.7
25/75	(−32.7) ^1^	36.6	18.6	19.4	81.3	−3.85	183.3	2.10	6.3
0/100	(−42.8) ^1^	35.7	22.2	17.3	-	-	-	-	-

^1^ *T*_g,LAO_ of MH-4scLAO/2CLO overlapped with *T*_m,CLO_.

**Table 3:** Tensile properties of PEU-3DLAO and PEU-3scLAO/2CLOs (100/0, 75/25, 50/50, 25/75 and 0/100).

Sample	Tensile Strength (MPa)	Tensile Modulus (MPa)	Elongation at Break (%)	Tensile Toughness (MJ·m^−3^)
PEU-3DLAO	42.9 ± 3.0	2214 ± 187	2.26 ± 0.32	0.62 ± 0.25
PEU-3scLAO/2CLO				
100/0	54.7 ± 3.6	2374 ± 262	2.98 ± 0.79	0.41 ± 0.24
75/25	13.3 ± 0.5	163 ± 16	66.8 ± 4.7	6.98 ± 0.69
50/50	3.37 ± 0.42	26.3 ± 2.0	92.3 ± 8.4	2.36 ± 0.46
25/75	11.3 ± 0.9	17.3 ± 2.5	378 ± 27	24.2 ± 3.3
0/100	10.4 ± 0.9	136 ± 13	726 ± 113	56.8 ± 10.9

## Supplementary Materials

The following are available online at www.mdpi.com/1996-1944/9/7/591/s1. Figure S1: The first heating DSC curves of H3LLAO, PEU-3DLAO, H3scLAO and PEU-3scLAO/2CLOs (100/0, 75/25, 50/50, 25/75 and 0/100); Figure S2: The second heating DSC curves of H3scLAO and PEU-3scLAO/2CLOs (100/0, 75/25, 50/50, 25/75 and 0/100).
