# Supplementary material for: Stereocomplexation in Copolymer Networks Incorporating Enantiomeric Glycerol-Based 3-Armed Lactide Oligomers and a 2-Armed ɛ-Caprolactone Oligomer
Source: Materials (Basel). 2016 Jul 19;9(7):591. doi: 10.3390/ma9070591 (PMC5456934; doi:10.3390/ma9070591)
Supplement: Supplementary file 1 [file materials-09-00591-s001.pdf]

Supplementary Materials: Stereocomplexation in Copolymer Networks Incorporating Enantiomeric Glycerol-Based 3-Armed Lactide Oligomers and a 2-Armed ε-Caprolactone Oligomer

Ayaka Shibita, Seina Kawasaki, Toshiaki Shimasaki, Naozumi Teramoto and Mitsuhiro Shibata

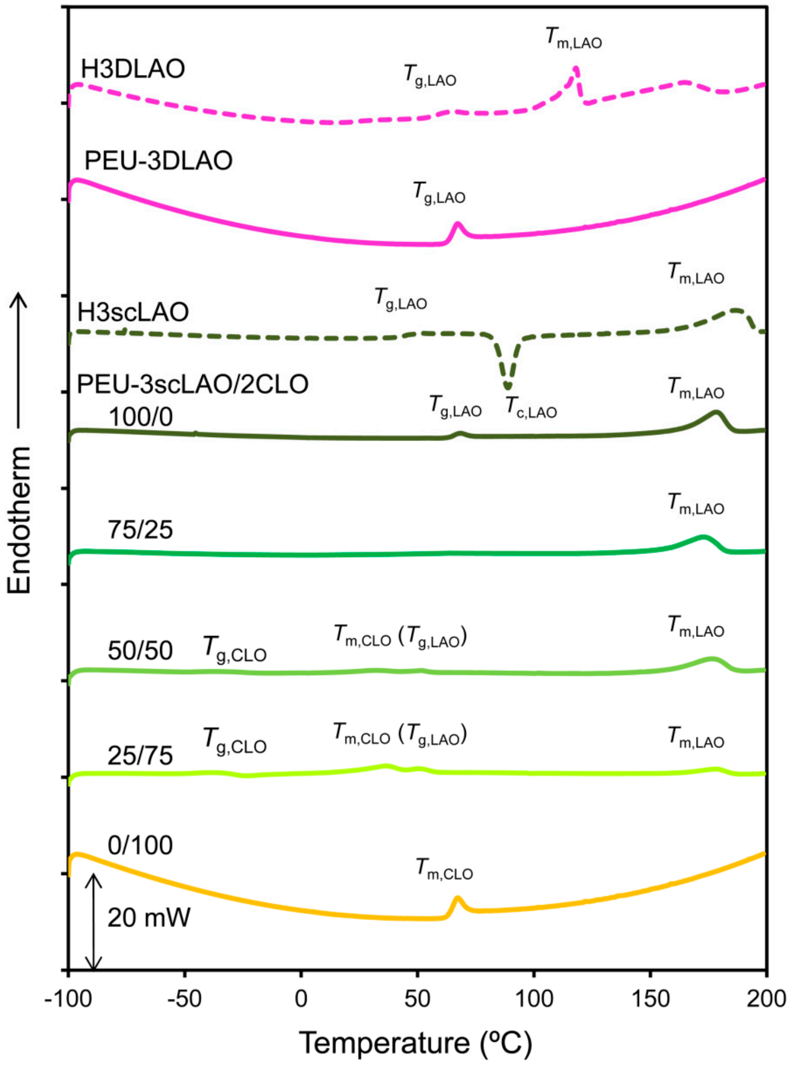

Figure S1. The first heating DSC curves of H3LLAO, PEU-3DLAO, H3scLAO and PEU-3scLAO/2CLOs (100/0, 75/25, 50/50, 25/75 and 0/100).

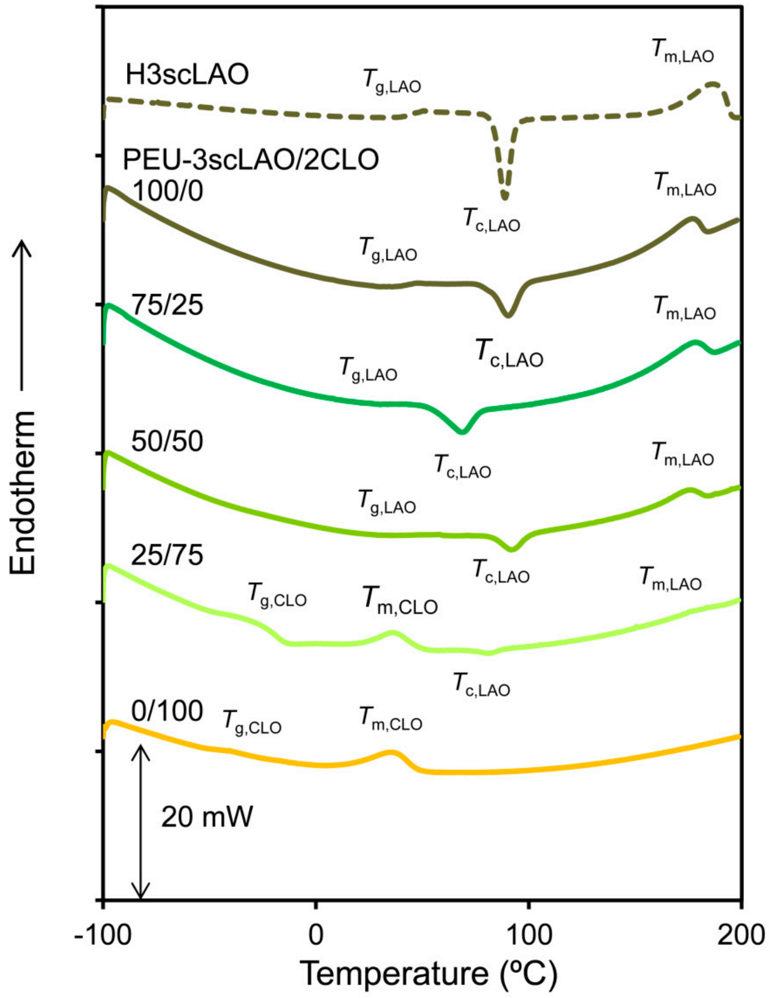

Figure S2. The second heating DSC curves of H3scLAO and PEU-3scLAO/2CLOs (100/0, 75/25, 50/50, 25/75 and 0/100).
